# Synthesis and anti-HSV-1 evaluation of new 3*H*-benzo[*b*]pyrazolo[3,4-*h*]-1,6-naphthyridines and 3*H*-pyrido[2,3-*b*]pyrazolo[3,4-*h*]-1,6-naphthyridines

**DOI:** 10.1186/2191-2858-2-3

**Published:** 2012-02-01

**Authors:** Alice MR Bernardino, Alexandre R Azevedo, Luiz CS Pinheiro, Júlio C Borges, Izabel CP Paixão, Milene Mesquita, Thiago ML Souza, Maurício S dos Santos

**Affiliations:** 1 Departamento de Química Orgânica, Instituto de Química, Programa de Pós-Graduação em Química, Universidade Federal Fluminense, Campus do Valonguinho, 24020-150, Niterói, RJ, Brazil; 2 Departamento de Biologia Celular e Molecular, Instituto de Biologia, Universidade Federal Fluminense, Campus do Valonguinho, 24020-150, Niterói, RJ, Brazil; 3 Programa de Pós-Graduação em Biologia Celular e Molecular, Fundação Oswaldo Cruz, Instituto Oswaldo Cruz, 21040-900, Rio de Janeiro, RJ, Brazil; 4 Departamento de Física e Química, Instituto de Ciências Exatas, Universidade Federal de Itajubá, 37500-903, Itajubá, MG, Brazil

**Keywords:** HSV-1, 1,6-naphthyridines, pyrazolonaphthyridines, heterocycles

## Abstract

**Background:**

Herpes simplex virus type-1 (HSV-1) is the primary cause of facial lesions (mouth, lips, and eyes) in humans. The widespread use of acyclovir and nucleoside analogues has led to emergence of HSV strains that are resistant to these drugs. Recently, non-nucleoside anti-HSV compounds have received considerable attention. 1,6-Naphthyridines are a class of heterocyclic compounds that exhibit a broad spectrum of biological activities such as inhibitor of HIV-1 integrase, HCMV, FGF receptor-1 tyrosine kinase, and the enzyme acetylcholinesterase. We previously reported the synthesis, SAR studies, and evaluation anti-HSV-1 activity of 3*H*-benzo[*b*]pyrazolo[3,4-*h*]-1,6-naphthyridines. In the course of our search for new 1,6-naphthyridines derivatives with potential activity against HSV-1, we have synthesized and evaluated new 3*H*-benzo[*b*]pyrazolo[3,4-*h*]-1,6-naphthyridines **(1a-k) **and 3*H*-pyrido[2,3-*b*]pyrazolo[3,4-*h*]-1,6-naphthyridines **(2a-c)**.

**Results:**

A known synthetic approach was used for preparing new 3*H*-benzo[*b*]pyrazolo[3,4-*h*]-1,6-naphthyridines **(1a-k) **and 3*H*-pyrido[2,3-*b*]pyrazolo[3,4-*h*]-1,6-naphthyridines **(2a-c)**, starting from ethyl 4-chloro-1-phenyl-1*H*-pyrazolo[3,4-*b*]pyridine-5-carboxylate **(7)**. All compounds were identified by FTIR, ^1^H NMR, and mass spectrometry. The antiviral effect on HSV-1 virus replication was determined.

**Conclusions:**

The compounds **1d**, **1f**, **1g**, and **1h **exhibited the highest anti-HSV-1 activity. In general, 3*H*-benzo[*b*]pyrazolo[3,4-*h*]-1,6-naphthyridines were more effective inhibitors than their corresponding 3*H*-pyrido[2,3-*b*]pyrazolo[3,4-*h*]-1,6-naphthyridines. The compound **1h **reduced the virus yield in 91% at 50 μM and exhibited a low cytotoxicity (CC_50 _600 μM).

## Background

Herpes simplex virus type-1 (HSV-1) is a large enveloped virus containing double-stranded DNA genomes of approximately 152 kb in size. HSV-1 is the primary cause of facial lesions (mouth, lips, and eyes) in humans [1,2]. Most of clinical anti-herpes virus compounds are nucleoside analogues, such as acyclovir (ACV), which is the most common drug used on treatment of HSV infections [3-5]. However, the widespread use of these compounds has been associated with the emergence of drug-resistant HSV strains [5]. The discovery of new non-nucleoside anti-HSV-1 agents with different mechanisms of action could offer an additional strategy against drug resistance of viruses. Several examples of non-nucleoside inhibitors have been proposed as candidate drugs for the treatment of herpes [3,6-11].

1,6-Naphthyridines are a class of heterocyclic compounds that exhibit a broad spectrum of biological activities such as inhibitor of HIV-1 integrase [12-15], HCMV [16,17], FGF receptor-1 tyrosine kinase [18], and the enzyme acetylcholinesterase [19]. Many routes for the syntheses of 1,6-naphthyridines derivatives have previously been reported [20-24].

**Figure 1:**
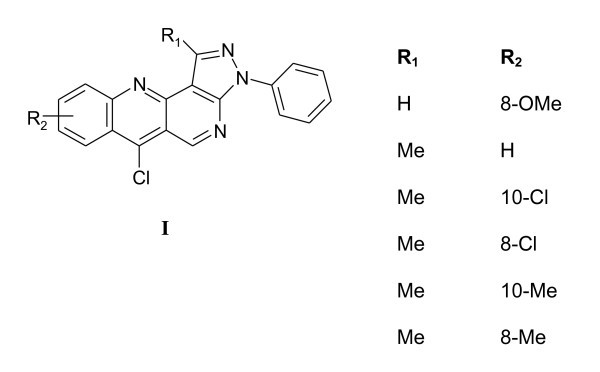
**Structure of 3*H*-benzo[*b*]pyrazolo[3,4-*h*]-1,6-naphthyridines I previously evaluated against HSV-1**.

**Scheme 1:**
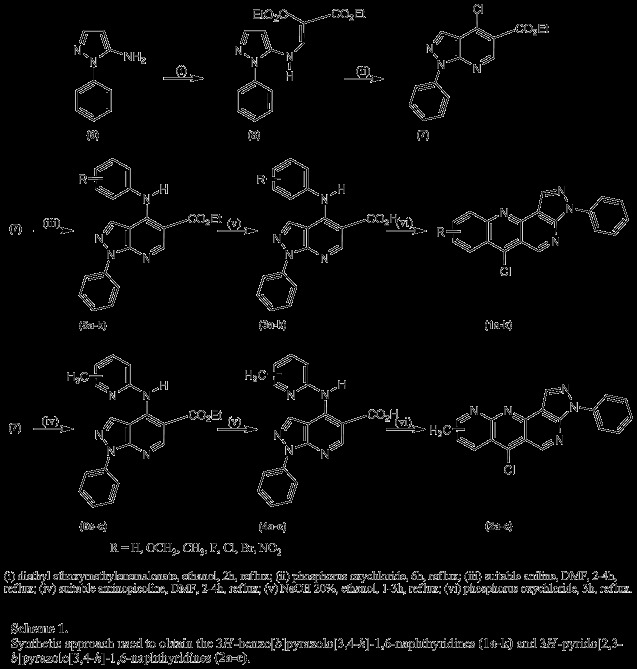
Synthetic approach used to obtain the 3*H*-benzo[*b*]pyrazolo[3,4-*h*]-1,6-naphthyridine derivatives (1a-k), and new three 3*H*-pyrido[2,3-*b*]pyrazolo[3,4-*h*]-1,6-naphthyridine derivatives (2a-c).

Recently, our research group reported the synthesis, SAR studies, and evaluation anti-HSV-1 activity of 3*H*-benzo[*b*]pyrazolo[3,4-*h*]-1,6-naphthyridines derivatives **I **(Figure 1) [25]. In the course of our search for new 1,6-naphthyridines derivatives with potential activity against HSV-1, we have synthesized and evaluated new 3*H*-benzo[*b*]pyrazolo[3,4-*h*]-1,6-naphthyridines **(1a-k) **and 3*H*-pyrido[2,3-*b*]pyrazolo[3,4-*h*]-1,6-naphthyridines **(2a-c) **(Scheme 1).

## Results and discussion

### Chemistry

A known synthetic approach was used for preparing the 3*H*-benzo[*b*]pyrazolo[3,4-*h*]-1,6-naphthyridines **(1a-k) **and 3*H*-pyrido[2,3-*b*]pyrazolo[3,4-*h*]-1,6-naphthyridines **(2a-c)**, starting from ethyl 4-chloro-1-phenyl-1*H*-pyrazolo[3,4-*b*]pyridine-5-carboxylate **(7) **(Scheme 1) [26-28]. In the first step, ethyl *α*-carboethoxy-*β*-(5-pyrazolylammonium)acrylate **(8) **was prepared by the condensation between 5-amino-1-phenyl-1*H*-pyrazole **(9) **and diethyl ethoxymethylenemalonate, in ethanol. The cyclization of the acrylate **8 **was carried out by refluxing in phosphorus oxychloride to afford 4-chloro1-phenyl-1*H*-pyrazolo[3,4-*b*]pyridine-5-carboxylate **(7) **in 75% yield [26-28]. Nucleophilic displacement of the chlorine atom in compound **7 **by aromatic amines gave ethyl 4-(arylamino)-1-phenyl-1*H*-pyrazolo[3,4-*b*]pyridine-5-carboxylates (**5a-k**) in yields 52-82% [26,29]. Similarly, aminopicolines were used to obtain ethyl 4-[(methylpyridin-2-yl)amino]-1-phenyl-1*H*-pyrazolo[3,4-*b*]pyridine-5-carboxylates **(6a-c) **in yields 50-60%. These were achieved by heating at 140°C without solvents for 2-4 h an equimolar mixture of the appropriate aniline or aminopicoline and the compound **7**. However, better results were obtained when these reactions were carried out in solvents such as DMF [25]. Subsequent hydrolysis of the esters **5a-k **and **6a-c **afforded the corresponding 4-(arylamino)-1-phenyl-1*H*-pyrazolo[3,4-*b*]pyridine-5-carboxylic acids (**3a-k**) and 4-[(methylpyridin-2-yl)amino]-1-phenyl-1*H*-pyrazolo[3,4-*b*]pyridine-4-carboxylic acids **(4a-c)**, in high yields, 86-93 and 80-93%, respectively [28]. For producing 3*H*-benzo[*b*]pyrazolo[3,4-*h*]-1,6-naphthyridines **(1a-k) **and 3*H*-pyrido[2,3-*b*]pyrazolo[3,4-*h*]-1,6-naphthyridines **(2a-c)**, the respective carboxylic acids **3a-k **and **4a-c **were cyclized with phosphorus oxychloride at 110°C over a period of 3 h [25,30]. The tetracyclic compounds **1a-k **and **2a-c **were isolated in 60-70% yield.

### Biological evaluation

The targets 3*H*-benzo[*b*]pyrazolo[3,4-*h*]-1,6-naphthyridines **(1a-k) **and 3*H*-pyrido[2,3-*b*]pyrazolo[3,4-*h*]-1,6-naphthyridines **(2a-c) **were evaluated for inhibition of HSV-1 replication in infected Vero cells. Results are shown in Table 1. Compounds **1d**, **1f**, **1g**, and **1h **exhibited the highest anti-HSV-1 activity. Compound **1h **reduced the virus yield in 91% at 50 μM. In general, 3*H*-benzo[*b*]pyrazolo[3,4-*h*]-1,6-naphthyridines **(1a-k) **were more effective inhibitors than their corresponding 3*H*-pyrido[2,3-*b*]pyrazolo[3,4-*h*]-1,6-naphthyridines **(2a-c)**.

**Table 1:** Anti-HSV-1 activity of 3*H*-benzo[*b*]pyrazolo[3,4-*h*]-1,6-naphthyridines (1a-k) and 3*H*-pyrido[2,3-*b*]pyrazolo[3,4-*h*]-1,6-naphthyridines (2a-c)

Compound	*R*	% of inhibition of virus yield (HSV-1)
**1a**	H	20,6
**1b**	9-OCH_3_	50,0
**1c**	9-CH_3_	68,0
**1d**	9-Cl	80,0
**1e**	8-Cl	60,0
**1f**	9-NO_2_	80,0
**1g**	8-NO_2_	87,0
**1h**	9-F	91,0
**1i**	8-F	65,0
**1j**	9-Br	30,0
**1k**	8-Br	30,0
**2a**	7-CH_3_	11,0
**2b**	8-CH_3_	ND
**2c**	9-CH_3_	65,0
**ACV**	-	96.0 ± 1.0

The experimental concentration of **1a-k **and **2a-c **was 50 μM and for ACV 10 μM.

Results are presented as the mean of triplicate experiments.

ACV has been included for comparison purposes.

Compounds with nearly the same antiviral effects were evaluated for cytotoxicity in Vero cells. EC_50 _and the selectivity index (SI) were determined in parallel. Several of the new compounds prevented the cytopathic effect of HSV-1 in Vero cells, at micromolar concentrations, and were minimally toxic to Vero cells resulting in a good SI. The MTT assay indicated that compound **1h **exhibited a low cytotoxicity (CC_50 _600 μM). Trypan blue and MTT showed similar results (data not shown). ACV results have been included for comparison purposes (Table 2).

**Table 2:** Anti-HSV-1 activity, cytotoxicity and SI in Vero cells for 6-chloro-3-phenyl-9-fluoro-3*H*-benzo[*b*]pyrazolo[3,4-*h*]-1,6-naphthyridine (1h)

Compounds	*R*	EC_50_^a ^(μM)	CC_50_^b ^(μM)	**S.I.**^**c**^
**1h**	9-F	0,07	600	8571
**ACV**		1.09 ± 0.25	960 ± 156	880

ACV has been included for comparison purposes.

^a^50% Effective concentration or concentration required to inhibit HSV-1 virus yield.

^b^50% Cytotoxic concentration or concentration required to reduce the viability of host cells by 50%.

^c^Selective index (CC_50_/EC50).

## Conclusions

In summary, a new series of 3*H*-benzo[*b*]pyrazolo[3,4-*h*]-1,6-naphthyridines **(1a-k) **and 3*H*-pyrido[2,3-*b*]pyrazolo[3,4-*h*]-1,6-naphthyridine **(2a-c) **were synthesized and some of them were potent anti-HSV-1 agents. The compounds **1d**, **1f**, **1g**, and **1h **exhibited the highest anti-HSV-1 activity, being the 3*H*-benzo[*b*]pyrazolo[3,4-*h*]-1,6-naphthyridine derivatives, in general, more effective inhibitors than their corresponding 3*H*-pyrido[2,3-*b*]pyrazolo[3,4-*h*]-1,6-naphthyridines. The compound **1h **reduced the virus yield in 91% at 50 μM and exhibited a low cytotoxicity (CC_50 _600 μM). The mechanism of antiviral activity of these compounds is under investigation.

## Experimental

Melting points were determined on a Fisatom 430D and are uncorrected. ^1^H NMR spectra were recorded on a Varian Unity Plus spectrometer for 300 MHz, with tetramethylsilane as the internal standard. Chemical shifts (δ) are reported in parts per million (ppm) and the coupling constants (J) in Hertz (Hz). Fourier transform infrared absorption spectra were recorded in a Perkin-Elmer Spectrum One FTIR spectrophotometer. The solid samples were measured using potassium bromide (KBr) pellets. Thin-layer chromatography was performed on Uniplates (silica gel). All chemicals were reagent grade. High-resolution mass spectral analysis was recorded using a Finingan MAT 711A.

### General procedures for the synthesis of 3*H*-benzo[*b*]pyrazolo[3,4-*h*]-1,6-naphthyridine derivatives (1a-k), and 3*H*-pyrido[2,3-*b*]pyrazolo[3,4-*h*]-1,6-naphthyridine derivatives (2a-c)

The key intermediate ethyl 4-chloro-1-phenyl-1*H*-pyrazolo[3,4-*b*]pyridine-5-carboxylate **(7) **was prepared according to literature [26-28]. An equimolar mixture of **7 **(4 mmol) and anilines or aminopicolines in 10 mL DMF was heated under reflux for 2-4 h. The reaction mixture, after cooling, was poured into 50 mL of ice-water. The precipitated was filtered, dried, and recrystallized from a mixture of ethanol and water. The compounds obtained **5a-k **and **6a-c **were reacted with 10 mL NaOH (20%) and 10 mL of ethanol under reflux for 1-3 h. On cooling to room temperature, the mixture was acidified with diluted hydrochloric acid (1:3), and the precipitate was filtered and recrystallized from DMF and water. A mixture of the acids **3a-k **and **4a-c **(1 mmol), and phosphorus oxychloride (5 mL) was heated under reflux for 3 h. The reaction mixture was inverted over crushed ice. In some cases the excess of phosphorus oxychloride was removed under reduced pressure before inverting over crushed ice and neutralized. The new compounds **1a-k **and **2a-c **were isolated in yields 60-70%. The resulting precipitate was collected and purified by flash column chromatography (FC, silica gel). The structures of the compounds were elucidated by FTIR, ^1^H NMR, and mass spectrometry.

### (1a) 6-chloro-3-phenyl-3*H*-benzo[*b*]pyrazolo[3,4-*h*]-1,6-naphthyridine

Yield 70%; mp 259-260°C; IR (KBr, cm^-1^) ν_max _C-H 3084, C = C 1596, C = N 1502; ^1^H NMR (DMSO-d6, 300 MHz) δ 8.92 (1H, s, H-1), 8.40 (1H, d, *J *= 8.1 Hz, H-10), 7.95 (1H, dd; *J *= 8.1 Hz, H-9), 7.70 (1H, dd, *J *= 8.1 Hz, H-8), 7.81 (1H, d, *J *= 8.1 Hz, H-7), 9.40 (1H, s, H-5), 8.33 (2H, d, *J *= 7.5 Hz, H-2', H-6'), 7.50 (2H, t, *J *= 7.5 Hz, H-3', H-5'), 7.52 (1H, t, *J *= 7.5 Hz, H-4'); EI (70eV) *m/z *(%): M^+ ^330.00761 (100).

### (1b) 6-chloro-3-phenyl-9-methoxy-3*H-*benzo[*b*]pyrazolo[3,4-*h*]-1,6-naphthyridine

Yield 68%; mp > 300°C; IR (KBr, cm^-1^) ν_max _C-H 3083, C = C 1595, C = N 1504; ^1^H NMR (DMSO-d6, 300 MHz) δ 8.99 (1H, s, H-1), 7.26 (1H, s, H-10), 7.78-7.56 (5H, m, H-3', H-4', H-5', H7, H-8), 9.36 (1H, s, H-5), 8.31 (2H, d, *J *= 8.0 Hz, H-2', H-6'), 4.07 (3H, s, Ar-OCH_3_); EI (70 eV) *m/z *(%): M^+. ^360.07037 (100).

### (1c) 6-chloro-3-phenyl-9-methyl-3*H*-benzo[*b*]pyrazolo[3,4-*h*]-1,6-naphthyridine

Yield 65%; mp > 300°C; IR (KBr, cm^-1^) ν_max _C-H 3083, C = C 1595, C = N 1503; ^1^H NMR (DMSO-d6, 300 MHz) δ 8.99 (1H, s, H-1), 7.79-7.57 (6H, m, H-3', H-4', H-5', H7, H-8, H-10), 9.42 (1H, s, H-5), 8.40 (2H, d, *J *= 8.4 Hz, H-2', H-6'), 1.39 (3H, s, Ar-CH_3_); EI (70eV) *m/z *(%): M^+. ^344.81022 (100).

### (1d) 6,9-dichloro-3-phenyl-3*H*-benzo[*b*]pyrazolo[3,4-*h*]-1,6-naphthyridine

Yield 60%; mp > 300°C; IR (KBr, cm^-1^) ν_max _C-H 3082, C = C 1598, C = N 1503; ^1^H NMR (DMSO-d6, 300 MHz) δ 9.08 (1H, s, H-1), 7.99-7.53 (6H, m, H-3', H-4', H-5', H7, H-8, H-10), 9.38 (1H, s, H-5), 8.41 (2H, d, *J *= 8.4 Hz, H-2', H-6'); EI (70 eV) *m*/*z *(%): M^+. ^364.01693 (100).

### (1e) 6,8-dichloro-3-phenyl-3*H*-benzo[*b*]pyrazolo[3,4-*h*]-1,6-naphthyridine

Yield 68%; mp > 300°C; IR (KBr, cm^-1^) ν_max _C-H 3084, C = C 1598, C = N 1503; ^1^H NMR (DMSO-d6, 300 MHz) δ 8.97 (1H, s, H-1), 7.87 (1H, d, *J *= 8.1 Hz, H-10), 7.89 (1H, d, *J *= 8.1 Hz, H-9), 8.22 (1H, s, H-7), 9.31 (1H, s, H-5), 8.27 (2H, d, *J *= 8.1 Hz, H-2', H-6'), 7.46 (2H, dd, *J *= 7.5 Hz, H-3', H-5'), 7.65 (1H, t, *J *= 7.5 Hz, H-4'); EI (70 eV) *m/z *(%): M^+. ^364.01789 (100).

### (1f) 6-chloro-3-phenyl-9-nitro-3*H*-benzo[*b*]pyrazolo[3,4-*h*]-1,6-naphthyridine

Yield 60%; mp > 300°C; IR (KBr, cm^-1^) ν_max _C-H 3084, C = C 1596, C = N 1503; ^1^H NMR (DMSO-d6, 300 MHz) δ 8.92 (1H, s, H-1), 8.84 (1H, s, H-10), 8.05 (1H, d, *J *= 7.5 Hz, H-8), 8.02 (1H, d, *J *= 7.5 Hz, H-7), 9.40 (1H, s, H-5), 8.31 (2H, d, *J *= 7.5 Hz, H-2', H-6'), 7.52 (2H, dd, *J *= 7.5 Hz, H-3', H-5'), 7.71 (1H, t, *J *= 7.5 Hz, H-4'); EI (70 eV) *m/z *(%): M^+. ^375.03743 (100).

### (1g) 6-chloro-3-phenyl-8-nitro-3*H*-benzo[*b*]pyrazolo[3,4-*h*]-1,6-naphthyridine

Yield 60%; mp 280-281°C; IR (KBr, cm^-1^) ν_max _C-H 3100, C = C 1592, C = N 1500; ^1^H NMR (DMSO-d6, 300 MHz) δ 8.90 (1H, s, H-1), 8.12 (1H, d, *J *= 7.8 Hz, H-10), 8.80 (1H, d, *J *= 7.8 Hz, H-9), 8.89 (1H, s, H-7), 9.42 (1H, s, H-5), 8.30 (2H, d, *J *= 7.5 Hz, H-2', H-6'), 7.51 (2H, dd, *J *= 7.5 Hz, H-3', H-5'), 7.68 (1H, t, *J *= 7.5 Hz, H-4'); EI (70 eV) *m/z *(%): M^+^. 375.70261 (100).

### (1h) 6-chloro-3-phenyl-9-fluoro-3*H*-benzo[*b*]pyrazolo[3,4-*h*]-1,6-naphthyridine

Yield 62%; mp 275-277°C; IR (KBr, cm^-1^) ν_max _C-H 3100, ν C = C 1600, ν C = N 1503; ^1^H NMR (DMSO-d6, 300 MHz) δ 9.34 (1H, s, H-1), 8.45 (1H, s, H-10), 7.83-7.62 (1H, m, H-8), 9.02 (1H, d, *J *= 8.4 Hz, H-7), 9.49 (1H, s, H-5), 8.20 (2H, d, *J *= 7.5 Hz, H-2', H-6'), 7.25 (2H, dd, *J *= 7.5 Hz, H-3', H-5'), 7.41 (1H, t, *J *= 7.5 Hz, H-4'); EI (70 eV) *m/z *(%): M^+^. 348.04188 (100).

### (1i) 6-chloro-3-phenyl-8-fluoro-3*H*-benzo[*b*]pyrazolo[3,4-*h*]-1,6-naphthyridine

Yield 65%; mp 278-279°C; IR (KBr, cm^-1^) ν_max _C-H 3051, C = C 1598, C = N 1503; ^1^H NMR (DMSO-d6, 300 MHz) δ 9.06 (1H, s, H-1), 8.03 (1H, d, *J *= 7.5 Hz, H-10), 8.05 (1H, m, H-9), 8.02 (1H, d, *J *= 8.4 Hz, H-7), 9.37 (1H, s, H-5), 8.32 (2H, d, *J *= 7.5 Hz, H-2', H-6'), 7.51 (1H, t, *J *= 7.5 Hz, H-4'), 7.69 (2H, dd, *J *= 7.5 Hz, H-3', H-5'); EI (70 eV) *m/z *(%): M^+^. 348.09473 (100).

### (1j) 9-bromo-6-chloro-3-phenyl-3*H*-benzo[*b*]pyrazolo[3,4-*h*]-1,6-naphthyridine

Yield 60%; mp > 300°C; IR (KBr, cm^-1^) ν_max _C-H 3052, C = C 1593, C = N 1503; ^1^H NMR (DMSO-d6, 300 MHz) δ 9.05 (1H, s, H-1), 8.09 (1H, s, H-10), 7.90-7.50 (5H, m, H-3', H-4', H-5', H7, H-8), 9.30 (1H, s, H-5), 8.30 (2H, d, *J *= 7.5 Hz, H-2', H-6'); EI (70 eV) *m/z *(%): M^+^. 409.96178 (100).

### (1k) 8-bromo-6-chloro-3-phenyl-3*H*-benzo[*b*]pyrazolo[3,4-*h*]-1,6-naphthyridine

Yield 68%; mp > 300°C; IR (KBr, cm^-1^) ν_max _C-H 3085, C = C 1596, C = N 1501; ^1^H NMR (DMSO-d6, 300 MHz) δ 8.95 (1H, s, H-1), 7.92-7.52 (5H, m, H-3', H-4', H-5', H7, H-10), 8.42 (1H, d, *J *= 8.4 Hz, H-9), 9.40 (1H, s, H-5), 8.30 (1H, d, *J *= 7.5 Hz, H-2', H-6'); EI (70 eV) *m/z *(%): M^+^. 409,67991 (100).

### (2a) 6-chloro-3-phenyl-7-methyl-3*H*-pyrido[2,3-*b*]pyrazolo[3,4-*h*]-1,6-naphthyridine

Yield 60%; mp 230-232°C; IR (KBr, cm^-1^) ν_max _C-H 3085, C = C 1596, C = N 1501; ^1^H NMR (DMSO-d6, 300 MHz) δ 8.72 (1H, s, H-1), 8.26 (1H, d, *J *= 7.5 Hz, H-9), 7.68 (1H, d, *J *= 7.5 Hz, H-8), 9.44 (1H, s, H-5), 8.26 (2H, dd, *J *= 7.5 Hz, H-2', H-6'), 7.50 (1H, t, *J *= 7.5 Hz, H-4'), 7.68 (2H, dd, *J *= 7.5 Hz, H-3', H-5'), 1.37 (3H, s, Ar-CH_3_); EI (70eV) *m/z *(%): M^+^. 345.74391 (100).

### (2b) 6-chloro-3-phenyl-8-methyl-3*H*-pyrido[2,3-*b*]pyrazolo[3,4-*h*]-1,6-naphthyridine

Yield 65%; mp 255-256°C; IR (KBr, cm^-1^) ν_max _C-H 3084, C = C 1596, C = N 1502; ^1^H NMR (DMSO-d6, 300 MHz) δ 8.72 (1H, s, H-1), 8.92 (1H, s, H-9), 7.91 (1H, s, H-7), 9.58 (1H, s, H-5), 8.42 (2H, d, *J *= 7.5 Hz, H-2', H-6'), 7.59 (1H, t, *J *= 7.5 Hz, H-4'), 7.78 (2H, dd, *J *= 7.5 Hz, H-3', H-5'), 1.42 (3H, s, Ar-CH_3_); EI (70 eV) *m/z *(%): M^+^. 345.78660 (100).

### (2c) 6-chloro-3-phenyl-9-methyl-3*H*-pyrido[2,3-*b*]pyrazolo[3,4-*h*]-1,6-naphthyridine

Yield 62%; mp 247-249°C; IR (KBr, cm^-1^) ν_max _C-H 3084, C = C 1596, C = N 1502; ^1^H NMR (DMSO-d6, 300 MHz) δ 8.83 (1H, s, H-1), 7.67 (1H, d, *J *= 7.5 Hz, H-8), 7.93 (1H, d, *J *= 7.5 Hz, H-7), 9.41 (1H, s, H-5), 8.39 (2H, d, *J *= 7.5 Hz, H-2', H-6'), 7.56 (1H, t, *J *= 7.5 Hz, H-4'), 7.75 (2H, dd, *J *= 7.5 Hz, H-3', H-5'), 1.39 (3H, s, Ar-CH_3_); EI (70 eV) *m/z *(%): M^+^. 345.76572 (100).

### Biological assays

Compounds were tested as inhibitor of HSV replication in Vero (African green monkey kidney, obtained from the American Type Culture Collection) cells. They were grown in DMEM (Gibco Laboratories) supplemented with 2% heat-inactivated fetal bovine serum (purchased from Fazenda Pig), 8% calf serum (purchased from Centro Pan-Americano de Febre Aftosa), 2.25% sodium bicarbonate, 500 U/mL penicillin, 100 μg/mL streptomycin, and 2.5 μg/mL amphotericin B. HSV-1 (ACR-29 strain) was kindly provided by Marcia Wigg (Universidade Federal do Rio de Janeiro, Brazil) and was routinely propagated in Vero cells. Virus stocks were stored at -70°C until use. ACV was purchased from Sigma (A 4669). It was dissolved in sterile deionized water and further diluted in culture medium. MTT was purchased from Sigma. Virus infectivity was measured by a dilution method using a 96-well microtitre plate and expressed as 50% tissue culture infections dose (TCID_50_). Cells grown in 96-well microtitre were inoculated with virus at input 1 PFU (plaque-forming unit)/cell for 2 h at 37°C. After virus adsorption, virus inoculum was replaced by a culture medium containing quinolone acyclonucleobases carboxylic acid and their correspondent esters at the concentration of 50 μM. Control cultures were incubated with media without compounds. After 3 days of incubation at 37°C in 5% CO_2 _atmosphere, the culture medium was harvested and the virus titre of each sample was determined in terms of 50% tissue culture dose (TCID 50/mL) by endpoint dilution.

### Cytotoxicity

The cytotoxicity of the compounds was tested in Vero cells using two methods, namely, MTT and trypan blue dye exclusion assay. Monolayers of uninfected cells were incubated with culture medium containing different concentrations of compounds for 72 h at 37°C. The medium was then removed, the cells trypsinized and viable cells counted by trypan blue dye exclusion test. The 50% cytotoxic concentration (CC_50_) was calculated by linear regression analysis of the dose-response curves generated from these data. In the second method, monolayer of Vero cells in 96-multiwell plates were incubated with MTT (5 μg/mL) at 37°C for 4 h. After this period, SDS 10% and 0.01 N HCl were added to each well and incubated overnight. The plates were read using an automatic plate reader with a 540-nm test wavelength and a 690-nm reference wavelength. Plaque reduction assay was performed utilizing Vero cells at a density of 3 × 10^5 ^infected with various dilutions of the supernatant from a yield reduction assay for 1 h at 37°C and 5% CO_2_. After adsorption, the plates were washed and the medium was replaced with DMEM containing methylcellulose 1% and fetal bovine serum 5%. After incubation for 72 h, the monolayers were fixed with 1% formaldehyde in PBS, methylcellulose removed, and cell stained with a 0.1% solution of crystal violet in 70% methanol. The virus yield assay was performed as follows. Confluent Vero cells were washed with PBS and infected with HSV-1 at moi of 1 PFU/cell for 1 h at 37°C. The infected cells were washed with PBS and covered with a culture medium containing either no compounds or a different concentration of compounds. 20 h after adsorption, cells were lysed by freezing and thawing (three times), and the supernatant consisting of culture medium and lysed cells was obtained by centrifugation at 400*g *for 10 min at 4°C. Virus titre was determined by the plaque assay in Vero cells as described above. Data were statistically analyzed by Student's *t*-test for a significance level of *p *< 0.05.

## Abbreviations

DMEM: Dulbecco's modified Eagle's medium; DMF *N*: *N*-dimethylformamide; HCMV: human cytomegalovirus; HSV-1: herpes simplex virus type-1; MTT: (3-(4,5-dimethylthiazol-2-yl)-2,5-diphenyl tetrazolium bromide; PBS: phosphate buffered saline; PFU: plaque-forming unit; SDS: sodium dodecyl sulphate.

## Competing interests

The authors declare that they have no competing interests.
